# Left hepatectomy with suprahepatic inferior vena cava resection and reconstruction under veno-arterial extracorporeal membrane oxygenation for intrahepatic cholangiocarcinoma: a case report

**DOI:** 10.1186/s40792-022-01468-9

**Published:** 2022-09-28

**Authors:** Shunichi Ariizumi, Masakazu Yamamoto, Azumi Hamasaki, Yoshihito Kotera, Takaaki Kato, Hiroto Egawa, Hiroshi Niinami, Goro Honda

**Affiliations:** 1 Department of Surgery, Institute of Gastroenterology, Tokyo Women’s Medical University, Tokyo, Japan; 2 Utsunomiya Memorial Hospital, Tochigi, Japan; 3 Department of Cardiovascular Surgery, Tokyo Women’s Medical University, Tokyo, Japan

## Abstract

**Background:**

Curative surgery is the most effective treatment for intrahepatic cholangiocarcinoma (ICC). When an ICC involves the suprahepatic inferior vena cava (IVC), hepatectomy with suprahepatic IVC resection and reconstruction is challenging. For reconstruction of the suprahepatic IVC, total hepatic vascular exclusion (THVE), veno-venous bypass, and/or in situ hypothermic portal perfusion are required, but mortality and morbidity remain high.

**Case presentation:**

We present a 73-year-old woman with mass-forming ICC which invaded the suprahepatic IVC and middle hepatic vein. Left hepatectomy, suprahepatic IVC resection, and reconstruction with an artificial graft were successfully performed during veno-arterial extracorporeal membrane oxygenation (V-A ECMO) to maintain blood pressure. While clamping the IVC diagonally, the right hepatic vein confluence could be preserved. No congestion in the right liver was seen; therefore, there was no requirement for the Pringle maneuver or THVE during reconstruction. No morbidity or mortality was seen after surgery.

**Conclusions:**

Hepatectomy with suprahepatic IVC resection and reconstruction under V-A ECMO can be performed safely. When an ICC invades the suprahepatic IVC, V-A ECMO during resection and reconstruction of the suprahepatic IVC with an artificial graft is recommended as one of the options.

## Introduction

Curative surgery is the most effective treatment for intrahepatic cholangiocarcinoma (ICC). When an ICC invades the inferior vena cava (IVC), resection and reconstruction of the IVC are required for curative surgery [1–4]. Simple tangential or segmental IVC resection and reconstruction can be performed without a veno-venous bypass (V-V bypass). However, an ICC involves the suprahepatic IVC and hepatic vein (HV) confluence, total hepatic vascular exclusion (THVE) is mostly required to resect and reconstruct the suprahepatic IVC with an artificial graft. For such complicated reconstruction of the suprahepatic IVC and HV-confluence, adequate reconstruction time due to V-V bypass, in situ hypothermic portal perfusion, and/or cardiopulmonary bypass are important in order to maintain systemic blood pressure. This report describes our experience with a successful suprahepatic IVC resection and reconstruction with an artificial graft using veno-arterial extracorporeal membrane oxygenation (V-A ECMO) in a patient with ICC.

## Case presentation

**Fig. 1:**
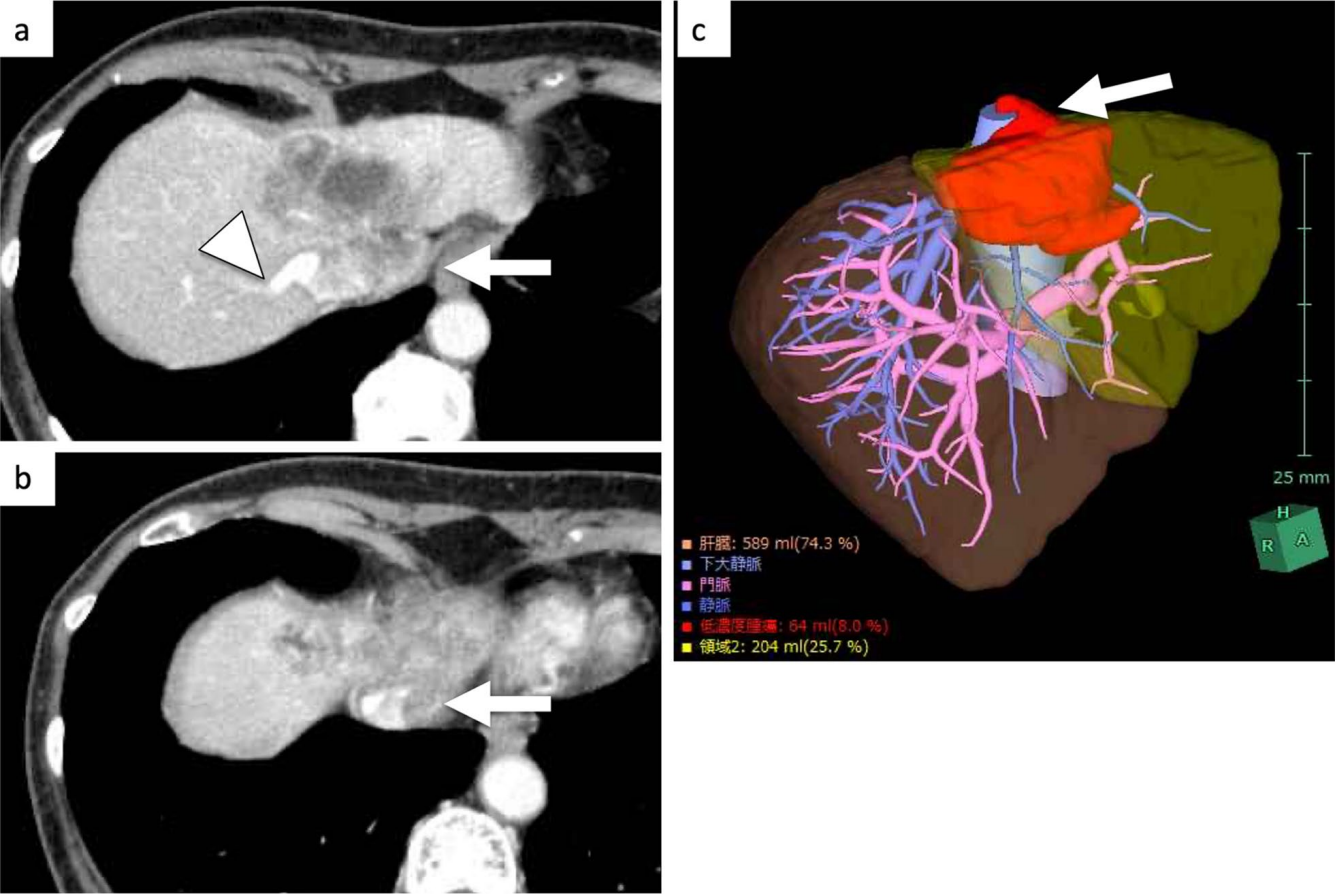
CT showed a mass-forming intrahepatic cholangiocarcinoma, 7 cm in diameter, in segment 4 (**a**). The ICC invaded the suprahepatic IVC, the MHV, and the LHV confluence (arrow, **a**–**c**), while the tumor did not invade the RHV (arrowhead, **a**)

**Fig. 2:**
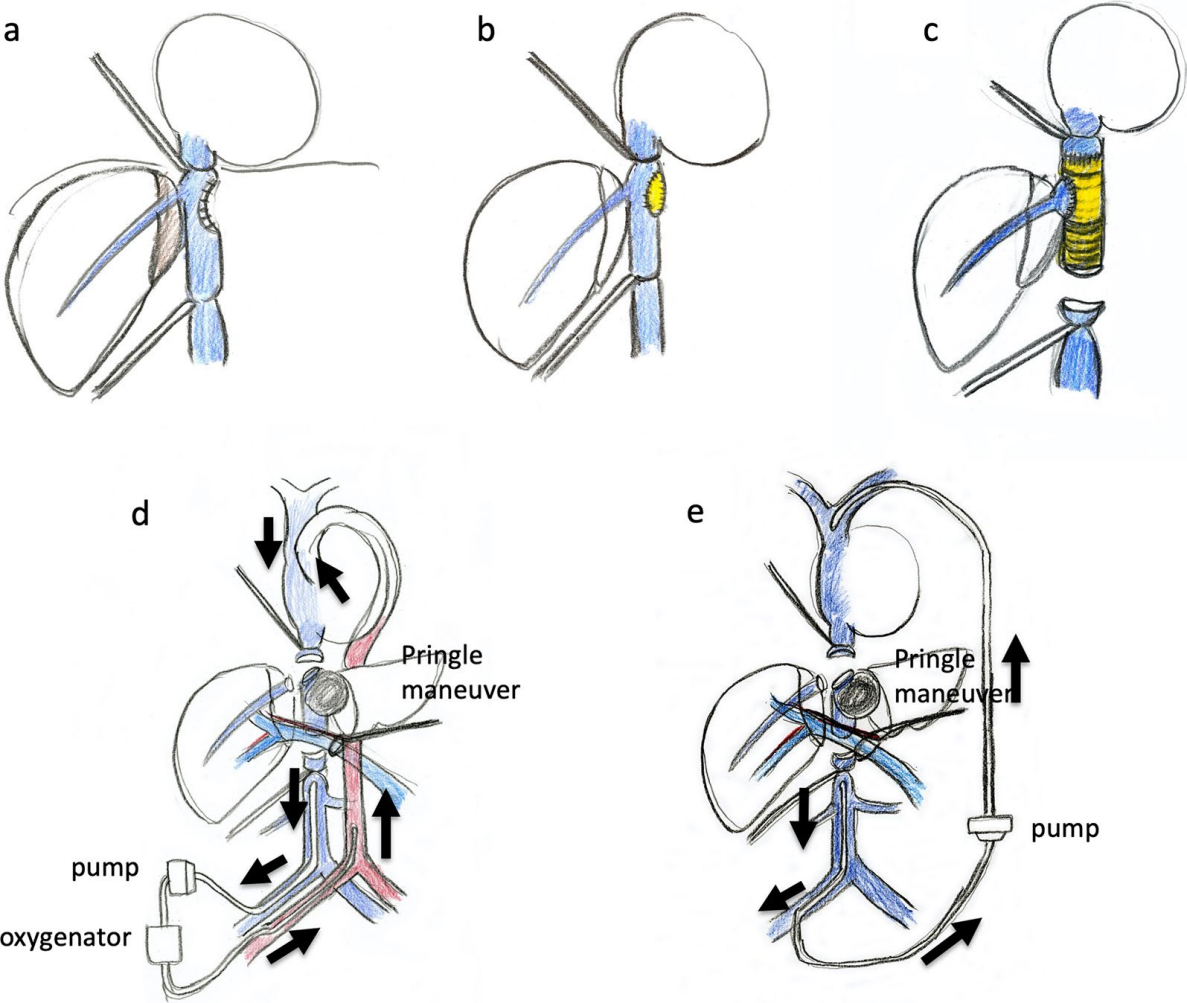
For reconstruction of the suprahepatic IVC and RHV with an artificial graft, we discussed and considered various types of reconstruction of the IVC (**a**–**c**) with V-A ECMO (**d**) or V-V bypass (**e**) in order to maintain blood pressure safely among cardiovascular surgeons, anesthesiologists, medical engineers, and gastroenterological surgeons

A 73-year-old woman presented with no symptoms after a liver tumor had been detected at an annual check-up. She had no past history of chronic hepatitis or blood transfusion. Admission laboratory tests revealed normal hemoglobin (12.4 g/dl), normal platelet counts (21.4 × 10^4^/μl), normal serum total bilirubin (0.7 mg/dl), normal serum albumin (4.4 g/dl), and normal serum prothrombin time (100%). The tumor markers AFP, CEA and CA19-9 were 4 ng/ml, 6.7 ng/ml and 35 U/ml, respectively. The Child–Pugh classification was A, and the indocyanine green retention rate at 15 min was 6%. Computed tomography (CT) scan showed a 7-cm ICC of the mass-forming type in segments 4 and 8. The ICC invaded the suprahepatic IVC, the middle HV (MHV), and the left HV (LHV) confluence, while the tumor did not invade the right HV (RHV) and inferior RHV (Fig. 1). 3D CT volumetry showed a left liver volume of 26% (Fig. 1). There was no distant metastasis nor lymph node metastasis. We planned to perform left hepatectomy, suprahepatic IVC and MHV confluence resection, and suprahepatic IVC resection. For reconstruction of the suprahepatic IVC and RHV with an artificial graft, we discussed and considered various types of reconstruction of the IVC with V-A ECMO or V-V bypass in order to maintain blood pressure safely and adequately among cardiovascular surgeons, anesthesiologists, medical engineers, and gastroenterological surgeons (Fig. 2).

**Fig. 3:**
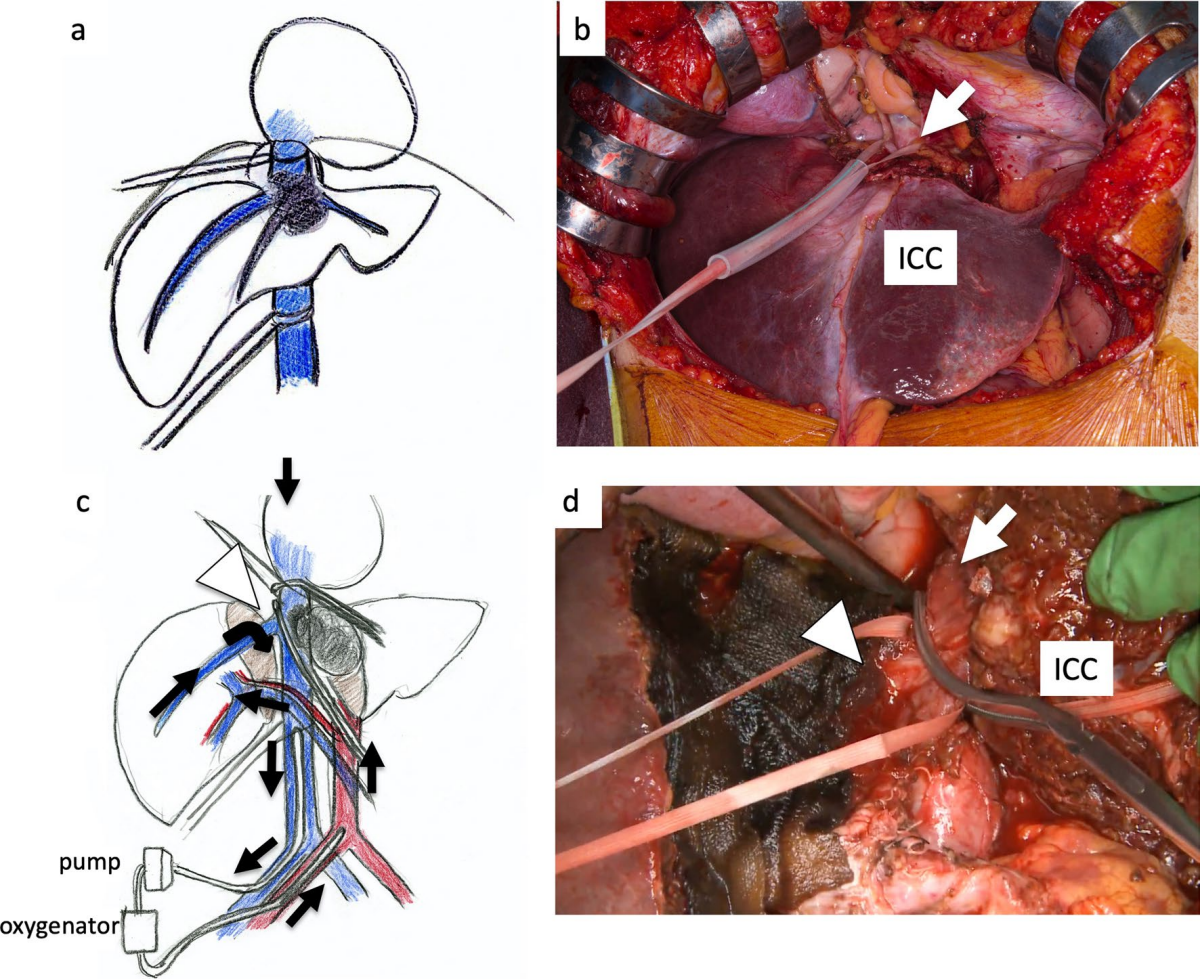
ICC invaded the MHV, LHV, suprahepatic IVC, and diaphragm. The suprahepatic IVC below the right atrium was taped after dissection of the diaphragm (arrow, **a**, **b**). Left hepatectomy including the suprahepatic IVC, MHV, LHV resection was performed (**c**, **d**). After dissection of the liver parenchyma, the IVC and RHV were confirmed by the anterior approach (arrowhead). For resection and reconstruction of the suprahepatic IVC, V-A ECMO was performed (**d**, arrow: blood flow during V-A ECMO)

**Fig. 4:**
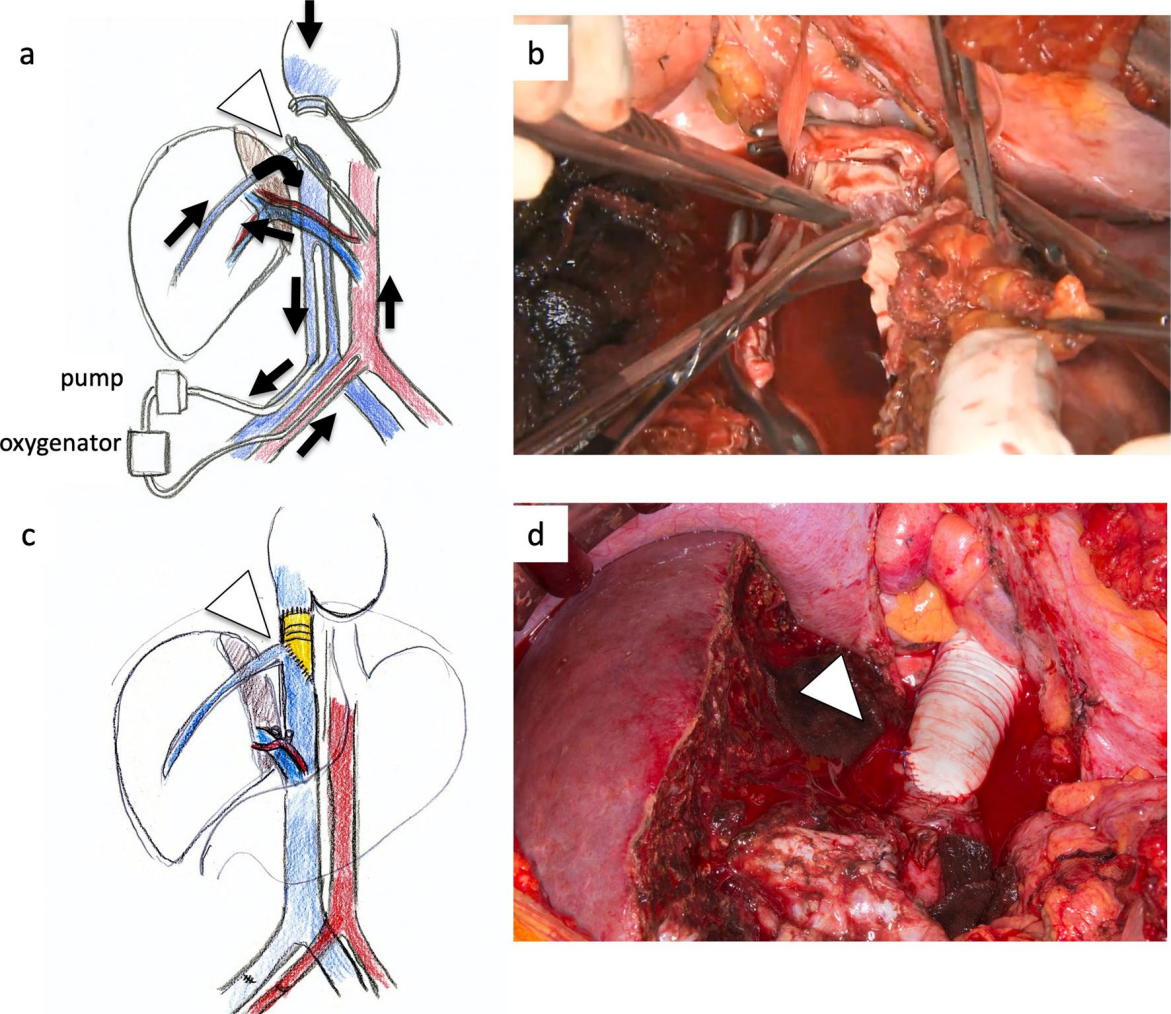
After clamping the IVC diagonally and preserving the root of the RHV (arrowhead), the suprahepatic IVC was resected and reconstructed with a ringed extended polytetrafluoroethylene tube graft (20 mm) (**a**–**d**). No congestion in the right liver was seen; therefore, there was no requirement for the Pringle maneuver or THVE (arrows: blood flow during V-A ECMO)

**Fig. 5:**
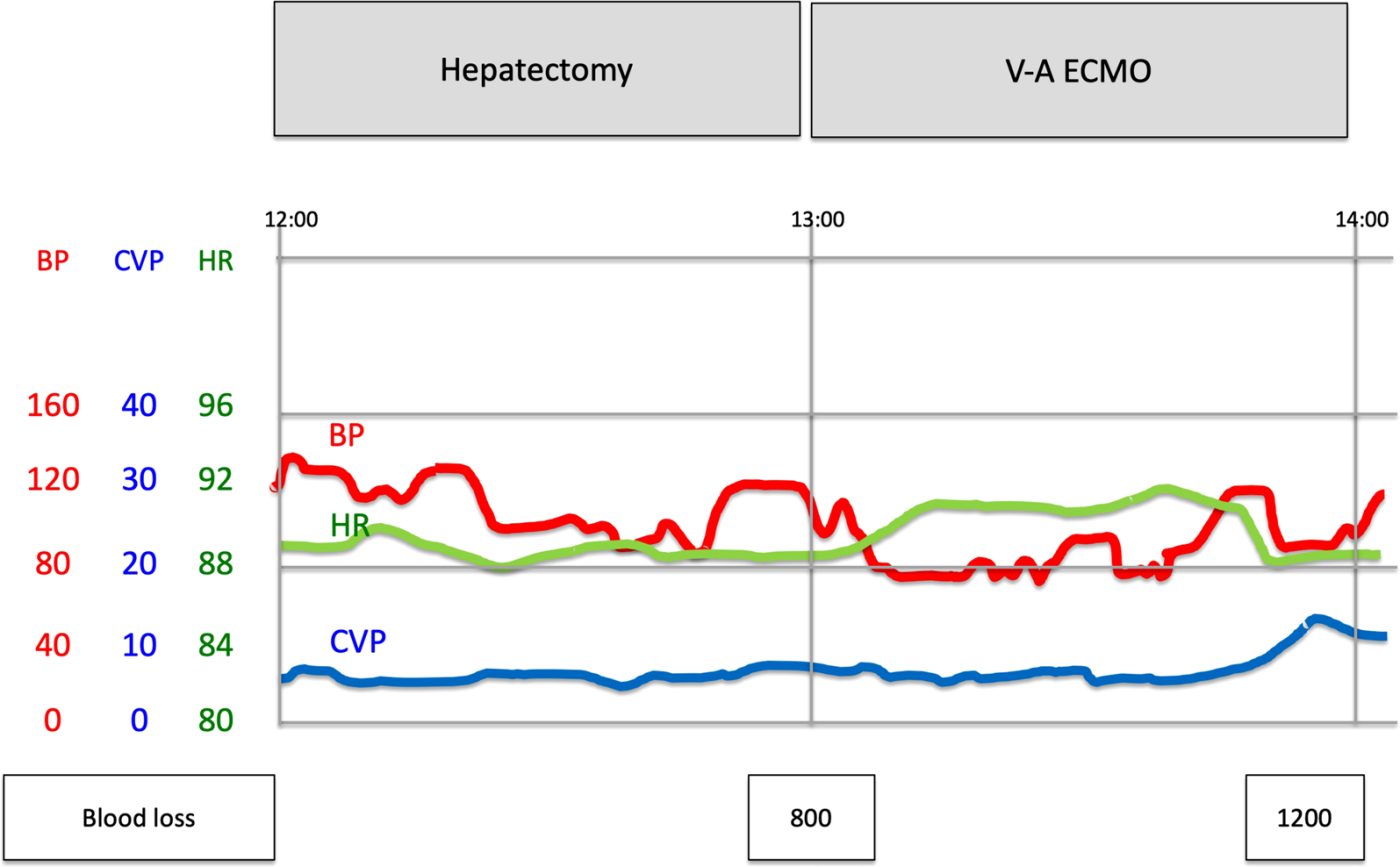
The vital signs and blood pressure were stable during hepatectomy and V-A ECMO (red: blood pressure)

**Fig. 6:**
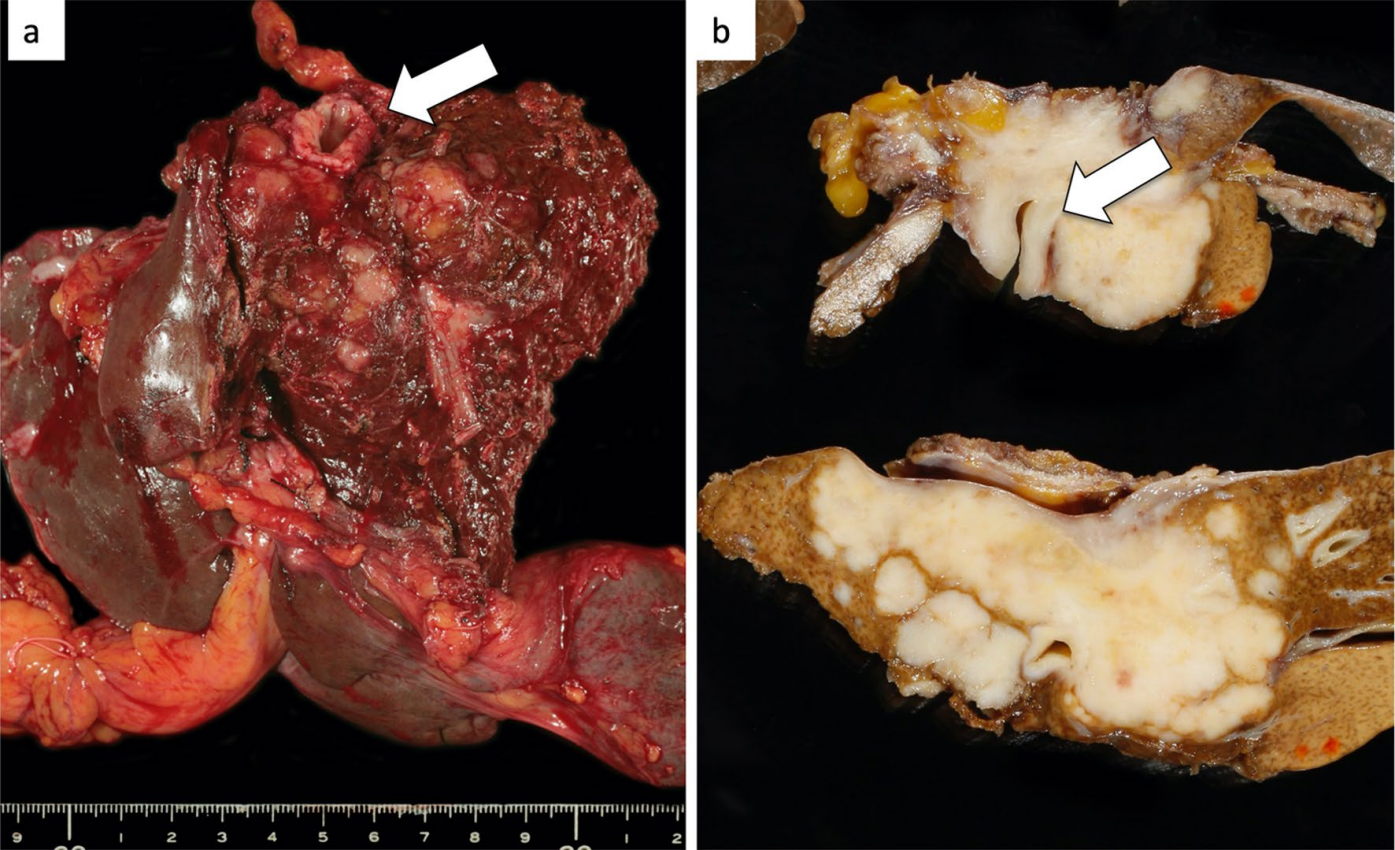
The tumor was of the mass-forming type macroscopically and cancer cells showed moderately to poorly differentiated ICC with invasion to the IVC, MHV, and diaphragm (arrow)

At first, laparotomy was done, and ICC in the left liver was confirmed. ICC invaded the MHV, LHV, suprahepatic IVC, and diaphragm (Fig. 2a). The suprahepatic IVC below the right atrium was taped after dissection of the diaphragm (Fig. 2b). Left hepatectomy including suprahepatic IVC, MHV, and LHV resection was performed. After ligation of the left hepatic artery and left portal vein, the liver parenchyma was transected under the Pringle maneuver and IVC clamping below the liver. After the IVC and the root of the RHV were confirmed by the anterior approach, V-A ECMO was performed (Fig. 2c, d). The V-A ECMO circuit was established via a 15 Fr cannula inserted into the right femoral artery and a 19.5 Fr cannula inserted into the right femoral vein. Activated clotting time (ACT) reached 350 s after intravenous injection of 5000 units of heparin sodium. Although the tumor invaded the left wall of the IVC, the RHV confluence was not involved. By clamping the IVC diagonally, the RHV confluence could be preserved (Figs. 2c, d, 3a, b). The suprahepatic IVC was resected and reconstructed with a ringed extended polytetrafluoroethylene tube graft (20 mm). No congestion in the right liver was seen; therefore, there was no requirement for the Pringle maneuver, THVE, or in situ hypothermic portal perfusion during reconstruction (Fig. 3c, d). The total IVC clamping time, the total V-A ECMO time, and the total operation time were 42 min, 54 min and 6 h 15 min, respectively. The vital signs and blood pressure were stable, and circulation was well maintained with the assistance of 1600 ml/min flow of V-A ECMO (Fig. 4). The total blood loss was 1200 ml (blood loss was 800 ml during hepatectomy and 400 ml during V-A ECMO). The tumor was of the mass-forming type macroscopically (Fig. 5) and cancer cells showed moderately to poorly differentiated ICC with invasion to the IVC, MHV, and diaphragm (Fig. 5). After surgery, laboratory tests revealed an elevated serum aspartate aminotransferase level (255 U/l), aspartate transaminase level (153 U/l), and serum total bilirubin (2.9 mg/dl) on postoperative day 1. The patient’s postoperative course was uneventful, and she was discharged 11 days after surgery. She is alive 14 months after surgery without local recurrence around the IVC. However, she underwent chemotherapy for intrahepatic metastases in the remnant liver (Fig. 6).

## Discussion

When an ICC invades the IVC, resection and reconstruction of the IVC are required for curative surgery [1–3]. Simple tangential or segmental IVC resection and reconstruction can be performed under clamping the IVC without V-V bypass [2, 3]. However, V-V bypass is sometimes required in order to maintain blood pressure after clamping of the IVC. For more complicated hepatectomy with suprahepatic IVC and HV-confluence resection and reconstruction, THVE is mostly required to reconstruct the suprahepatic IVC with an artificial graft [3]. For such complicated surgical procedures, adequate reconstruction time is required in order to maintain systemic blood pressure. In our present case, V-A ECMO was performed safely to maintain blood pressure during suprahepatic IVC resection and reconstruction with an artificial graft. While clamping the IVC diagonally, the RHV confluence could be preserved. Therefore, no THVE was performed. Suprahepatic IVC resection and reconstruction with an artificial graft under V-A ECMO is considered an option for ICC with suprahepatic IVC.

Higher operative morbidity and mortality rates have been reported after hepatectomy with IVC resection and reconstruction. Hemming et al. reported an operative mortality of 9% and operative morbidity of 43% in 60 patients with various liver cancers who underwent hepatectomy with IVC resection and reconstruction [4]. According to their report, overall 1- and 5-year survival rates were 89% and 35%, respectively. Azoulay et al. reported that among 22 patients who underwent combined liver resection with IVC reconstruction for liver metastasis, ICC or HCC, THVE was performed in 12 patients and V-V bypass in 12 patients [2]. Operative mortality was 4.5%, operative morbidity 64%, and 5-year survival rate 38.3% [2]. Tomimaru et al. reviewed 13 studies with 111 patients who underwent IVC resection and reconstruction, and reported that operative mortality was 8.1% [1]. According to these reports, hepatectomy with IVC resection was recommended if it was resectable despite the ICC invading the IVC [1].

Hepatectomy with resection and reconstruction of the suprahepatic IVC and HV confluences is challenging because more complicated reconstruction with an artificial graft is required [3]. For such complicated reconstruction of the suprahepatic IVC and HV-confluence, THVE, V-V bypass, and/or cardiopulmonary bypass are important in order to maintain systemic blood pressure [2, 3]. Furthermore, open heart surgery in order to suture between the right atrium and IVC is required [2, 5]. Azoulay et al. reported that patients should be selected carefully because of high mortality and morbidity rates; a 90-day mortality rate of 20% and morbidity rate of 70%, in 77 patients who underwent liver resection using THVE, V-V bypass, and in situ hypothermic portal perfusion [2]. In our present case, hepatectomy with suprahepatic IVC resection and reconstruction under V-A ECMO was successfully performed. While clamping the IVC diagonally, the RHV confluence could be preserved. Therefore, no THVE was performed. Preserving the RHV could prevent ischemic or congestive change of the remnant right liver; therefore, no morbidity or mortality was seen in our present case.

A V-V bypass during hepatectomy with IVC resection and orthotopic liver transplantation can be easily accessed from the femoral vein to the axillar vein or jugular vein. However, an inadequate bypass due to insufficient blood removal causes trouble maintaining blood pressure. Imperfect circulating blood volume and hypotension due to low flow through the bypass (collapse or kinking of the tubing or hypovolemia) during orthotopic liver transplantation with V-V bypass have been reported [6, 7]. Adequate reconstruction time and safe bypass are, therefore, required for complicated reconstruction of the suprahepatic IVC. Whang et al. reported a case of ICC with invasion to the suprahepatic IVC treated with left hepatectomy and suprahepatic IVC resection under cardiopulmonary bypass (CPB) [5]. They concluded that the use of CPB was effective and that a team approach between HBP and cardiothoracic surgeons was important [5]. Recently, the usefulness of V-A ECMO for not only cardiogenic shock patients, but also severe COVID-19 patients have been known.

V-A ECMO can maintain blood pressure during several days or months easily and adequately [8–10]. During V-A ECMO, cardiac output decreases because venous return volume decreases according to the pump flow volume. However, blood flow from V-A ECMO supplements the amount of perfusion, thus maintaining systemic circulation. Similarly, pulse pressure decreases during V-A ECMO; however, mean arterial pressure is maintained adequately. In our present case, hepatectomy with suprahepatic IVC resection and reconstruction could be performed safely under V-A ECMO by collaboration of cardiovascular and HBP surgeons.

V-V bypass does not require heparinization during surgery because of the heparin-coated circuit. However, V-A ECMO requires heparinization during surgery in order to prevent thrombosis. In our present case, heparin for V-A ECMO was administered after hepatectomy. Therefore, there was no morbidity, mortality, or uncontrollable bleeding due to the heparinization (blood loss was 800 ml during hepatectomy and 400 ml during V-A ECMO). Hepatectomy before heparinization can prevent uncontrollable bleeding during V-A ECMO with heparinization.

## Conclusions

This is the first English-language report on hepatectomy with suprahepatic IVC resection and reconstruction under V-A ECMO. For complicated reconstruction of the suprahepatic IVC, V-A ECMO is recommended as one of the options in order to maintain blood pressure safely and adequately.

## Data Availability

The authors declare that all the data in this article are available within the article.

## Abbreviations

ICC: Intrahepatic cholangiocarcinoma
IVC: Inferior vena cava
HV: Hepatic vein
CT: Computed tomography
THVE: Total hepatic vascular exclusion
V-V bypass: Veno-venous bypass
V-A ECMO: Veno-arterial extracorporeal membrane oxygenation
MHV: Middle hepatic vein
LHV: Left hepatic vein
RHV: Right hepatic vein
ACT: Activated clotting time
CPB: Cardiopulmonary bypass
